# Differences in sorption behavior of the herbicide 4-chloro-2-methylphenoxyacetic acid on artificial soils as a function of soil pre-aging

**DOI:** 10.1007/s11368-012-0550-9

**Published:** 2012-07-03

**Authors:** Georg Waldner, Wolfgang Friesl-Hanl, Georg Haberhauer, Martin H. Gerzabek

**Affiliations:** 1 Health and Environment Department, AIT Austrian Institute of Technology GmbH, Konrad-Lorenz-Strasse 24, 3430 Tulln, Austria; 2 Institute of Soil Research, University of Natural Resources and Life Sciences, Peter-Jordan-Strasse 82, 1190 Vienna, Austria

**Keywords:** Artificial soils, FTIR, Incubation time, MCPA, Soil components, Sorption

## Abstract

**Purpose:**

The sorption behavior of the herbicide 4-chloro-2-methylphenoxyacetic acid (MCPA) to three different artificial soil mixtures was investigated. Artificial soils serve as model systems for improving understanding of sorption phenomena.

**Materials and methods:**

The soils consisted of quartz, ferrihydrite, illite, montmorillonite, and charcoal. In a previous study, several selected mixtures had been inoculated with organic matter, and microbial aging (incubation) had been performed for different periods of time (3, 12, and 18 months) before conducting the sorption experiments. The effect of this pre-incubation time on the sorption behavior was determined. Interaction of MCPA with soil surfaces was monitored by aqueous phase sorption experiments, using high-performance liquid chromatography/ultraviolet and in selected cases Fourier-transformed infrared spectroscopy.

**Results and discussion:**

The sorption behavior showed large differences between differently aged soils; Freundlich and linear sorption model fits (with sorption constants *K*_*f*_, 1/*n* exponents, and *K*_*d*_ values, respectively) were given for pH = 3 and the unbuffered pH of ∼7. The largest extent of sorption from diluted solutions was found on the surfaces with a pre-incubation time of 3 months. Sorption increased at acidic pH values.

**Conclusions:**

Regarding the influence of aging of artificial soils, the following conclusions were drawn: young artificial soils exhibit stronger sorption at lower concentrations, with a larger *K*_*f*_ value than aged soils. A correlation with organic carbon content was not confirmed. Thus, the sorption characteristics of the soils are more influenced by the aging of the organic carbon than by the organic carbon content itself.

## Introduction

Transport, transformation, and fate of organic chemicals in soils and in the environment are topics of numerous investigations and reviews (Barrow 2008; Haberhauer et al. 2000; Barrow 1998; Calvet 1989). Retention may cause a reduced bioavailability but also increased problems with detoxification strategies; sorption of herbicides depends on various properties of soils and the substance itself. Soil properties depend on the one hand on the surface and structural characteristics of its inorganic components (minerals) like quartz (QU; Scheffer and Schachtschabel 2002), montmorillonite (MO; Li et al. 2003), illite (IL; Polubesova and Nir 1999), ferrihydrite (FH; Janney et al. 2000; Friesl-Hanl et al. 2009; Waldner et al. 2010; Klepsch et al. 2011), and on the other hand on the surface properties of organic matter–in our case charcoal (CH; Rennert et al. 2008; Derylo-Marczewska et al. 2010), and manure (MA; Haberhauer et al. 2001; Sheng et al. 2001). In a previous study (Heister et al. 2012; Pronk et al. 2012), several selected mixtures had been inoculated with organic matter, and microbial aging (incubation) had been performed for different periods of time (3, 12, and 18 months) before conducting the sorption experiments. The effect of this pre-incubation time on the sorption behavior was now investigated in the present study.

Each component shows its own specific sorption behavior (Totsche et al. 2010), which may or may not dominate the overall behavior of the incubated mixture, depending on the mixing ratios and/or extent of soil–organic interactions. QU exhibits almost no surface reactivity. MO is an expandable 2:1 clay mineral with a huge surface (predominantly inner surface) and is negatively charged at pH values commonly occurring in most soils. IL is also negatively charged but offers no inner surface. FH shows a huge surface and is positively charged at neutral pH values. CH is recalcitrant organic matter with a huge surface dominated by functional groups like carboxyl and graphene-like polycyclic aromatic surface structures. MA is a typical soil amendment and fertilizer, maintaining soil organic matter levels and fostering microbial activity; the manure used in the preparation of the soil mixtures was autoclaved. Artificial soil mixtures are a means to enhance understanding of the behavior of complex soil matrices and their interaction with xenobiotics like pesticides or heavy metals. The preparation of the artificial soils employed in this investigation is described elsewhere (Pronk et al. 2012).

4-Chloro-2-methylphenoxy acetic acid (MCPA) is an acidic herbicide belonging to a larger class of phenoxy acetic acid herbicides, which are widely used in agriculture, controlling annual and perennial weeds in cereals, grassland, trees, and turf. These herbicides represent a potential risk for water sources as they are poorly biodegradable and quite mobile in aqueous systems because of their acidic carboxyl group. MCPA has been found in well water in some countries and is classified by the US EPA as a potential groundwater contaminant and systemic toxicant (US EPA 1984). Due to the acidic carboxyl group (Addorisio et al. 2010), phenoxy acetic herbicides are able to form relatively strong complexes with polar species or, in anionic form, they can be involved in the formation of strong complexes with cations present in the soil. Cho et al. (2006) showed enhanced sorption of MCPA on activated carbon and a higher sorption of the herbicide at pH 3.5 and a decrease of sorption with increasing pH of the solution.

Still, the behavior of MCPA in soils needs further research, as it is a currently used herbicide and especially in order to understand the contribution of different soil components and their associations (i.e., newly formed soil organic matter, SOM, or humic acid–clay complexes) to MCPA sorption. However, due to the relatively simple chemical structure, MCPA is also a good model for sorption experiments. The specific aim of the present paper was to study the influence of different microbial aging periods of the artificial soil surfaces as well as of pH on sorption of MCPA as well as to check the applicability of sorption study concepts to previous studies with real soils. The novelty of this study is based on the specific properties of the artificial soils and the changes in sorption characteristic as a result of SOM formation and/or formation of humic acid–clay complexes.

## Materials and methods

### Chemicals

Technical grade (97 %) MCPA was used as model substance. Its solubility in water at 25 °C is 825 mg L^−1^ and its acidity constant (pK_*a*_) is 3.07. The agronomic dose of MCPA is between 1 and 2.5 kg ha^−1^.

Artificial soils were produced at TU Munich (Heister et al. 2012; Pronk et al. 2012) to obtain simple model soils of known composition and better-defined interfaces. These soils consisted of varying mixtures of QU, FH, CH, and IL. The mixtures had been amended with organic material (manure, MA) and inoculated with a soil microbial community for a certain time (3, 12, and 18 months, in the following labeled as 3 M, 12 M, and 18 M). The soils contained 42 % sand, 52 % silt, and 6 % of particles <6.3 μm of varying composition of aforementioned components. Further properties are reported in Table 1.

**Table 1:** Properties of artificial soil mixtures

		pH	pzc	SSA bulk/m²/g	SSA (<20 μm)/m²/g	Fe_d / mg/g	Fe_o / mg/g	C_org_ / mg/g		
Soil	Mineral composition	All incubation times		*t* = 0	3M	All incubation times	All incubation times	3 M	12 M	18 M
C	QU, FH, MA	6.8–7.7		2.7	6.8	5–6	0.7–0.9	16	15	13
E	QU, MO, CH, MA	6.9–7.6		2.5	7.3	–	–	30	28	26
H	QU, IL, FH, CH, MA	7–7.6		3.4	6.7	6	0.8–1.0	31	28	–
Individual components	MO		8.2	28.8						
	IL		8.3	12.3						
	CH			11.1						
	FH		7.9	200–400						

Soil data from Pronk (personal communication, 2010) and Pronk et al. (2012); BET data of components, own measurements; BET of FH (six lines) and pzc, literature data (Cornell and Schwertmann 1996)

*SSA* specific surface area of two fractions, bulk soil vs. fraction <20 μm; *C*_*org*_ organic carbon content; *Fe_d* dithionite-extractable fraction (total iron content); *Fe_o* oxalate-extractable fraction (poorly crystalline iron oxides); *3M etc.* incubation time (months)

Specific surface area (SSA) was measured by N_2_ sorption (BET isotherm). Soil C consisted of QU, FH, and MA; soil E was based on QU, MO, CH, MA, and soil H on QU, IL, FH, CH, and MA.

### Experimental design

Sorption isotherms were measured as follows: 6 g of each artificial soil sample was weighed into 30 mL centrifuge tubes made of glass. Six milliliters of 0.01 M CaCl_2_ aqueous solutions of MCPA containing initial pesticide concentrations ranging between 0.1 and 300 mg L^−1^ was added to each vessel. The flasks were shaken for 24 h. Preliminary kinetic experiments had demonstrated that sorption equilibrium was reached within less than 24 h. Before conducting the present sorption experiments, the soils had not been sterilized, i.e., microbial degradation was not excluded; it can only be neglected within the first 24 h comparing the duration of these experiments with typical half-lives under similar conditions (∼13 days; Smith 1982). A 0.01 M solution of CaCl_2_ was used to ease phase separation and to keep ionic strength similar to natural soil solutions. The experiments were carried out at 20 °C. After centrifuging, the supernatant equilibrium solution was removed and the concentrations of MCPA in this supernatant solution, *C*_eq_, were determined by high-performance liquid chromatography with an ultraviolet detector (Agilent 1100 series). As mobile phase, a mixture of 70 % methanol, 29.7 % H_2_O, and 0.3 % acetic acid was used (isocratic elution). The analysis was done on a Supelco Discovery HS C18 column (5 μm) at 25 °C and a flow of 1 mL min^−1^. The MCPA was determined at the wavelength of maximum absorption (282 and 228 nm at the lowest concentrations). The MCPA adsorbed, *C*_*s*_, was calculated from the difference between the initial and final concentration of the CaCl_2_ solution. Blanks containing no MCPA were included, and flasks containing only MCPA and no soil were analyzed to determine adsorption on the surface of the flasks. All sorption experiments were done at least in duplicate. The unbuffered and unadjusted natural pH was ∼7; additionally, in selected cases, HCl was added to adjust the pH to ∼3 (not checked after equilibration).

Fourier transform infrared spectra (FTIR) were recorded in transmission mode with a Perkin Elmer Spectrophotometer (System 2000) over a range of 4,000–400 cm^−1^. For spectroscopic characterization, 1 mg of sample were thoroughly mixed with 300 mg KBr (FTIR grade) and pressed to a pellet.

## Results and discussion

Linear isotherms were obtained for the initial very low concentration range. Linear distribution coefficients (*K*_*d*_ values) were obtained from the initial slope (first two data points) of the linear graph of *c*_*s*_ against *c*_eq_.

1 $$ {c_s} = {K_d}{c_{\text{eq}}} $$

- *c*_*s*_ … sorbed amount of substance on the solid surface (mg kg ^-1^)
- *c*_eq_ … solution equilibrium concentration (mg L ^-1^)
- *K*_*d*_ … sorption (distribution) coefficient [(mg kg ^-1^)/(mg L ^-1^)]
- *K*_oc_ = *K*_*d*_/(*f*_oc_), with *f*_oc_ = fraction of organic carbon

At higher, i.e., more realistic concentrations, or when the concentration spans several orders of magnitude, the Freundlich equation is more suitable for fitting the sorption behavior: sorption isotherms were thus also analyzed using the Freundlich equation and represented in a double-logarithmic plot:

2 $$ {c_s} = {K_f}{c_{\text{eq}}}^{{1/n}} $$

- in the linearized form: $\log \,{c_s} = \log \,{K_f} + 1/n\,\log \,{c_{{eq}}}$
- *K*_*f*_ … Freundlich constant [mg^1 − 1/*n*^ kg ^-1^ L^1/*n*^]; more intuitively: *K*_*f*_ = *c*_*s*_ at *c*_eq_ = 1 mg L^−1^
- 1/*n* … Freundlich exponent (slope of the double-log plot, or deviation from linearity in the linear form)

**Fig. 1:**
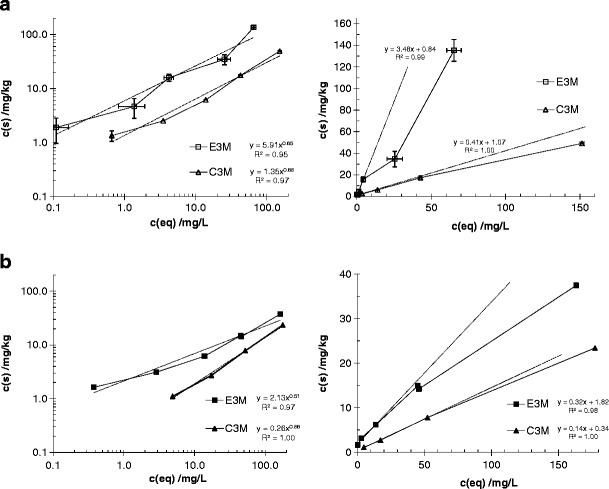
Sorption isotherms of MCPA on soils C vs. E after 3 months of incubation; effect of pH = 3 (**a**) vs. unadjusted and unbuffered pH (**b**, ∼7); *left* double logarithmic plot, *right* linear plot

A typical shape of sorption isotherms is represented in Fig. 1 and discussed below.

### The influence of pH on the adsorption of MCPA

Sorption experiments were performed at pH = 3 and at the unadjusted pH of ∼7. Upon acidification, an increase of sorption was observed; there is a clear shift of the sorption curves to the upper left (compare Fig.1 b with Fig.1 a, for both soils C and E, respectively). Freundlich parameters (*K*_*f*_ and 1/*n*) and linear distribution coefficients *K*_*d*_ as well as *K*_oc_ values are presented in Table 2. The *K*_oc_ is the sorption coefficient normalized to the organic carbon content; the values are not significantly different in most cases, except for the measurements at pH = 3, where the values are up to one order of magnitude larger.

**Table 2:** Freundlich sorption fits

pH→	7						3					
Soil	*K*_*f*_	1/*n*	*R*²	*K*_*d*_	*f*_oc_	*K*_oc_	*K*_*f*_	1/*n*	*R*²	*K*_*d*_	*f*_oc_	*K*_oc_
C3M	0.26	0.86	1.00	0.14	0.016	8.8	1.35	0.68	0.97	0.41	0.016	26
C12M					0.015						0.015	
C18M	0.16	0.97	0.98		0.013						0.013	
E3M	2.13	0.51	0.97	0.32	0.030	11	5.91	0.65	0.95	3.48	0.030	116
E12M					0.028						0.028	
E18M	0.35	0.85	0.99		0.026						0.026	
H3M	2.06	0.41	0.88	0.18	0.031	5.8					0.031	
H12M	0.54	0.81	1.00	0.41	0.028	15	7.9	0.68	0.99		0.028	
H18M	0.28	0.96	0.97	0.51			10.7	0.57	0.99			

Explanations cf. Eqs. 1 and 2; soil codes, *C3M* soil C, 3 months of pre-incubation, etc.; *f*_oc_ values calculated from Pronk et al. (2012)

*K*_*f*_ Freundlich constant, *1/n* exponent, *R²* coefficient of determination, *K*_*d*_ distribution coefficient (linear fit), *K*_*oc*_ *K*_*d*_/*f*_oc_

The lipophilic aromatic part of MCPA is not influenced by the pH, in contrast to the carboxylic group. The pK_*a*_ value of MCPA (pK_*a*_ = 3.07) implies that the compound exists mainly in the deprotonated anionic form throughout a wide pH range in natural waters and soil solutions. The results obtained indicated the greatest sorbed amount of MCPA at pH = 3 (where ∼50 % are present as deprotonated (anionic) and protonated (neutral) species, respectively). Ionic interaction of oppositely charged species is stronger than Van der Waals interactions; this is especially important in the discussion of the present case, considering the pK_*a*_ value of MCPA and the point of zero charge (pzc) of many of the soil components, e.g., iron oxides, montmorillonite, and illite: pzc > 8 (Scheffer and Schachtschabel 2002). At pH values between the pK_*a*_ and pzc, the mainly present anionic solution species (deprotonated MCPA in the carboxylate form) strongly interact with positively charged surface sites, whereas charcoal offers mainly uncharged surface sites and preferably sorbs neutral solution species (e.g., the neutral, i.e., protonated MCPA). This trend of increasing sorption of acidic herbicides with decreasing pH, as expected from theory, was observed by many authors like Haberhauer et al. (2001), Paszko (2011), and Hiller et al. (2012); besides, Paszko (2011) distinguished between hydrophobic and hydrophilic sorption of MCPA. The surface charge of the present soils was not determined here but can be expected to be net positive, at pH values below the pzc of the predominant soil components (e.g., Iglesias et al. 2010).

### Influence of soil composition and complexity

With increasing complexity of the soils (compare soils C vs. E and H after 3 months of pre-incubation, Fig. 1a and b, as well as Table 2), *K*_*f*_ values increased and the linearity decreased, as shown by the 1/*n* values falling below 0.5. However, in this case, the *K*_*d*_ values did not show a clear trend; the discrepancy between *K*_*f*_ and *K*_*d*_ values was also discussed by Karnjanapiboonwong et al. (2010). The charcoal content was expected to play a crucial role; there is a slight indication of its influence at both pH values in Fig. 1 (shift to the upper left, comparing soil C vs. E). The measured BET values are not that high (cf. new data in Table 1), so its influence is not expected to overrule any other contributions of other components. As a consequence, *K*_oc_ data did not show a clear trend, except for the drastically increased values at pH = 3.

### The influence of biological aging of artificial soils on the sorption of MCPA

**Fig. 2:**
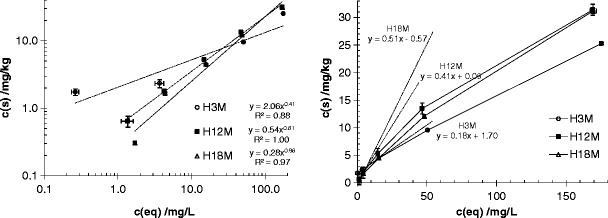
Sorption isotherms of MCPA on soil H after different incubation periods; unadjusted and unbuffered pH (∼7)

Typical sorption isotherms showed a shape like the one depicted in Fig. 2; additional information is given in Table 2. The obtained Freundlich and linear sorption fits are summarized for both pH values; for explanations cf. Eq. 1 and thereafter. The youngest artificial soil exhibits significant sorption at lowest concentrations; the most prominent difference seems to be a change in sorption characteristics upon aging.

Although the fit of the Freundlich isotherm is not always perfect—especially for soil H (3 months)—it still seems to be the most reasonable model, as all other sorption theories give worse fits (e.g., Langmuir); the linear sorption model is applicable only at low concentrations.

*K*_*f*_ decreases with increased soil aging, with a concomitant increase in the Freundlich exponent 1/*n*, whereas, according to the linear sorption model, the sorption capacity at low concentrations increases (shown by the increasing *K*_*d*_ value), with a substantial decrease at higher concentrations. Upon acidification, sorption capacity increases as well: both *K*_*f*_ and *K*_*d*_ values increase compared to their values at pH = 7, and at the same time the 1/*n* exponent does not show a clear trend.

The results of N_2_-BET measurements of each component of the soils are given in Table 1. It is obvious that the sum of the components would give a much larger specific surface area for sorption reactions than the incubated artificial soils (compare the larger values of SSA of MO, IL, and CH with the very small one of soils C, E, or H). The reduction of the surface area after incubation of the components might be due to interactions of reactive surface and therefore a blocking of reactive sites. Incubation of the artificial soils further reduced the reactivity of the surfaces for sorption with time, probably due to the interaction of the components and their associations, achieving stable micro-aggregates (Six et al. 2004). These micro-aggregates are the basis for the formation of macro-aggregates due to exudates of microorganisms like fungal hyphae or bacteria.

### Measurement of sorption by FTIR (detection of surface species)

**Fig. 3:**
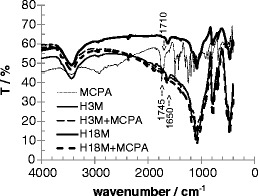
FTIR measurements of MCPA (*dashed thin line*) and soils H in different aging states and adsorbate systems

FTIR spectra of MCPA, soil H after 3- and 18-month incubation is shown in Fig. 3, along with the soils plus sorbed MCPA after a simulated sorption experiment: in the latter case, soil was impregnated with a solution of MCPA; the solution was not removed but evaporated. Subsequently, a KBr pellet with highly MCPA loaded soil sample was prepared. Nevertheless, the concentration was probably below the limit of detection to show any trace of residual or sorbed MCPA on the soil. In contrast to Pusino et al. (1995), the adsorbed herbicide could not be detected with FTIR. Yet, the most prominent features of the spectra of both pure MCPA and soils can be distinguished, i.e., the bands of carboxylic and carbonyl groups are located around 1,745 cm^−1^ (MCPA dimer) and 1,710 cm^−1^ (monomer), and 1,650 cm^−1^ in soils.

## Conclusions

Aging of artificial soils due to microbial activity results in varying sorption isotherms for MCPA. Older soils (with a longer pre-incubation time) show lower *K*_*f*_ and larger 1/*n* values closer to 1 (increased slope in the double-log plot). The Freundlich equation (monolayer sorption on heterogeneous surfaces) is sufficient to explain many of the sorption curves, with substantial deviations for the younger artificial soils (3 months of pre-incubation).

Regarding the influence of aging, the following conclusions can be drawn: in our experiments, young soils exhibit stronger sorption at lower concentrations, with a decreasing *K*_*f*_ and increasing *K*_*d*_ values compared to aged soils. Upon aging, the amount of sorbed species per unit surface area is more concentration-dependent. Thus, aging of the organic carbon influences the sorption behavior of the soils more than the organic carbon content itself. Yet, a correlation with organic carbon content is expected but cannot be derived from the present data. Further examination of the behavior of the aged soils and more data points regarding the evolution of the organic carbon content, e.g., monitoring SOM quality, would be required.

Some of the presented observations (e.g., increase of sorption upon acidification and a change of sorption characteristics with increased aging) are known from literature on real soils, but now these concepts were shown to be equally well applicable to artificial soils.

Using FTIR (KBr pellets, transmission mode), the concentration of sorbed species on the solid surface is below the limit of detection under the given circumstances.
